# Relevance of Subcutaneous Fat Thickness as a Risk Factor for Surgical Site Infections in Abdominal Surgeries

**DOI:** 10.7759/cureus.20946

**Published:** 2022-01-04

**Authors:** Ravikumar Teppa, Nandkishor Sopanrao Sude, Venkata Pavan Kumar Karanam, Bhaskara Veera Prasad Mallipudi

**Affiliations:** 1 General Surgery, Employees State Insurance Corporation Medical College and Hospital (ESICMCH), Hyderabad, IND; 2 General Surgery, Krishna Institute of Medical Sciences (KIMS), Hyderabad, IND

**Keywords:** drain, laparotomy, surgical site infection, thickness, subcutaneous fat

## Abstract

Introduction

Incisional surgical site infection is an important cause of postoperative morbidity which results in extended hospital stay and may result in future incisional hernia. We intended to evaluate the thickness of subcutaneous fat with a cut-off value of 2.5cm as a risk factor in causing surgical site infection using a simple, cost-effective, and direct intraoperative method for measuring subcutaneous fat thickness.

Methods

A total of 147 patients who underwent abdominal surgeries from September 2017 to April 2019 were included in this prospective study. A proforma was used to collect information of all patients regarding various variables. Abdominal subcutaneous fat thickness was measured in the supine position intraoperatively with a measuring scale from below dermis to rectus sheath at 1cm caudal to umbilicus level.

Results

The study's overall incidence of incisional surgical site infection (SSI) in laparotomy surgeries was 10.8%. Subcutaneous fat thickness was independently associated with incisional SSI. Subcutaneous fat thickness association with SSI was more statistically significant than that of BMI. The other associated risk factors were found to be obesity, diabetes, and emergency surgery.

Conclusion

Our results suggest that the risk of incisional SSI increases with the increased subcutaneous fat thickness of more than 2.5cm. Placement of subcutaneous drain in patients undergoing laparotomy with increased subcutaneous fat thickness plays a significant role in reducing the incidence of surgical site infection. Risk of SSI increases in obesity, diabetes, increased age group, dirty surgery, and emergency surgeries. Subcutaneous fat thickness is an independent risk factor for surgical site infection and subcutaneous drain decreases the risk of SSI in thick subcutaneous fat.

## Introduction

Surgical site infections (SSIs) are infections of the incision or organ or space that occur after surgery [1]. Surgical site infection (SSI), still continues to be one of the major sources of postoperative morbidity especially in low- and middle-income countries [2]. SSI results in prolonged hospitalization and the need for prolonged wound care, even at times requiring advanced methods of wound closure like negative pressure wound therapy. This results in diminished quality of life and poses a considerable economic burden to the patients [3,4].

In spite of widespread awareness regarding preventive strategies like refined surgical techniques, sterile operating environment, antibiotic prophylaxis, SSI still remains to be the frequent type of healthcare-associated infection. Although various initiatives like National Nosocomial Infections Surveillance (NNIS) System, National Healthcare Safety Network (NHSN), and Surgical Care Improvement Project (SCIP) provided insights into various risk factors associated with SSI, some factors are yet to be addressed in detail. Several studies have shown that body mass index does not account for body fat distribution [5,6]. Intuitively, local subcutaneous fat thickness serves as a better marker than body mass index in predicting surgical site infections especially in patients undergoing abdominal surgeries. Soper et al. reported that only patients with depth of subcutaneous fat > 3cm developed SSI after undergoing abdominal hysterectomy [7]. Fujii et al. reported a cut-oﬀ value for subcutaneous fat thickness (SFT) of 20mm in Asian patients who developed incisional surgical site infection (iSSI) after undergoing elective colorectal surgery [8]. Several previous studies have suggested the use of preoperative CT scan or USG to measure the thickness of subcutaneous fat (TSF) [8-12]. However, a CT scan is a costly procedure and carries a risk of unnecessary radiation exposure. Ultrasound is operator-dependent and prone to subjective variation.

This study hypothesized subcutaneous fat thickness (cut-off value: 2.5cm) as a more relevant factor than the body mass index in predicting surgical site infection in abdominal surgeries. We used a simple, cost-effective, and direct intraoperative method for measuring SFT. In addition, we also studied various other determinants and risk factors associated with SSI. This study will hopefully help to understand the factors which are associated with SSI and help in the clinical practice of surgery.

## Materials and methods

This is a prospective observational study conducted at a tertiary care hospital in Hyderabad, India, from July 2017 to April 2019. It was approved by the KIMS Ethics Committee for Thesis (#KIMS/EC/2017/10-07). Adults of age group 16-90 years undergoing elective and emergency abdominal surgeries were included in this study. SSI in our study included superficial (skin and subcutaneous tissue) and deep space (fascia and muscle) infections. Exclusion criteria were age less than 16 years and more than 90 years, organ space infections occurring more than 30 days after surgery. The sample size was calculated based on a previous study that recorded the prevalence of SSI as 14-16% among hospital inpatients [13]. Lower prevalence was considered to yield more samples. With 95% confidence interval (CI) and permissible error of 5%, the minimum sample size was calculated as n = 128. The total number of patients included in this study was 147.

Aims and objectives

The study aimed to study and analyze the determinants and risk factors associated with SSI. The primary objective was to evaluate the thickness of subcutaneous fat as a risk factor in causing surgical site infection in the following combinations: (a) thickness less than 2.5cm without drain, (b) thickness more than 2.5cm without drain, and (c) thickness more than 2.5cm with drain. The secondary objective was to evaluate the effect of other variables like type of surgery (emergency vs elective surgery), diabetes mellitus, obesity, age of the patient, and type of surgical wound on the incidence of SSI.

Procedural methodology

All the surgeries were done under universal aseptic conditions with a laminar flow operation theatre. No wound protector system was used for the surgeries. For clean wounds, the standard procedure for skin preparation was followed using the application of three coats of povidone-iodine to the skin, followed by a compulsory waiting period of 20 minutes until the skin dried up preceding surgical incision. For wounds other than clean wounds, povidone-iodine scrub was applied as three coats, followed by three coats of spirit and repeat three coats of povidone-iodine again with an intervening period of 15-20 minutes was uniformly followed preceding skin incision. All patients uniformly received prophylactic antibiotic dosage before incision. Standard antibiotics used for perioperative SSI prevention in our hospital were ceftriaxone preoperatively and postoperatively in combination with metronidazole. The standard time of prophylactic administration of antibiotic was just before anesthetic induction preoperatively in the operation theatre and postoperatively within 30 minutes of wound closure. SSI in our study was defined as superficial SSI if skin and subcutaneous tissue were involved and deep space SSI if fascia and muscle were also involved. Wound infection was suspected if there was a serous, non-purulent, purulent discharge with or without signs of inflammation (edema, redness, raised local temperature, induration, fever >38°C) at the wound site, or visible wound dehiscence. The surgical wound was inspected on an alternate day at the time of dressing and in case of suspicion of wound infection daily dressing and followed until complete wound healing occurred or the patient was discharged from the hospital.

Interventions and methods of measurement of the outcome of interest

Investigations relevant to the study were done which included hemoglobin levels, random blood sugar, blood urea, and serum creatinine levels, pus culture, sensitivity, and ultrasound of abdomen when indicated. A proforma was used to collect information from all patients. Each of the objectives was studied as follows.

Subcutaneous Fat Thickness

**Figure 1:**
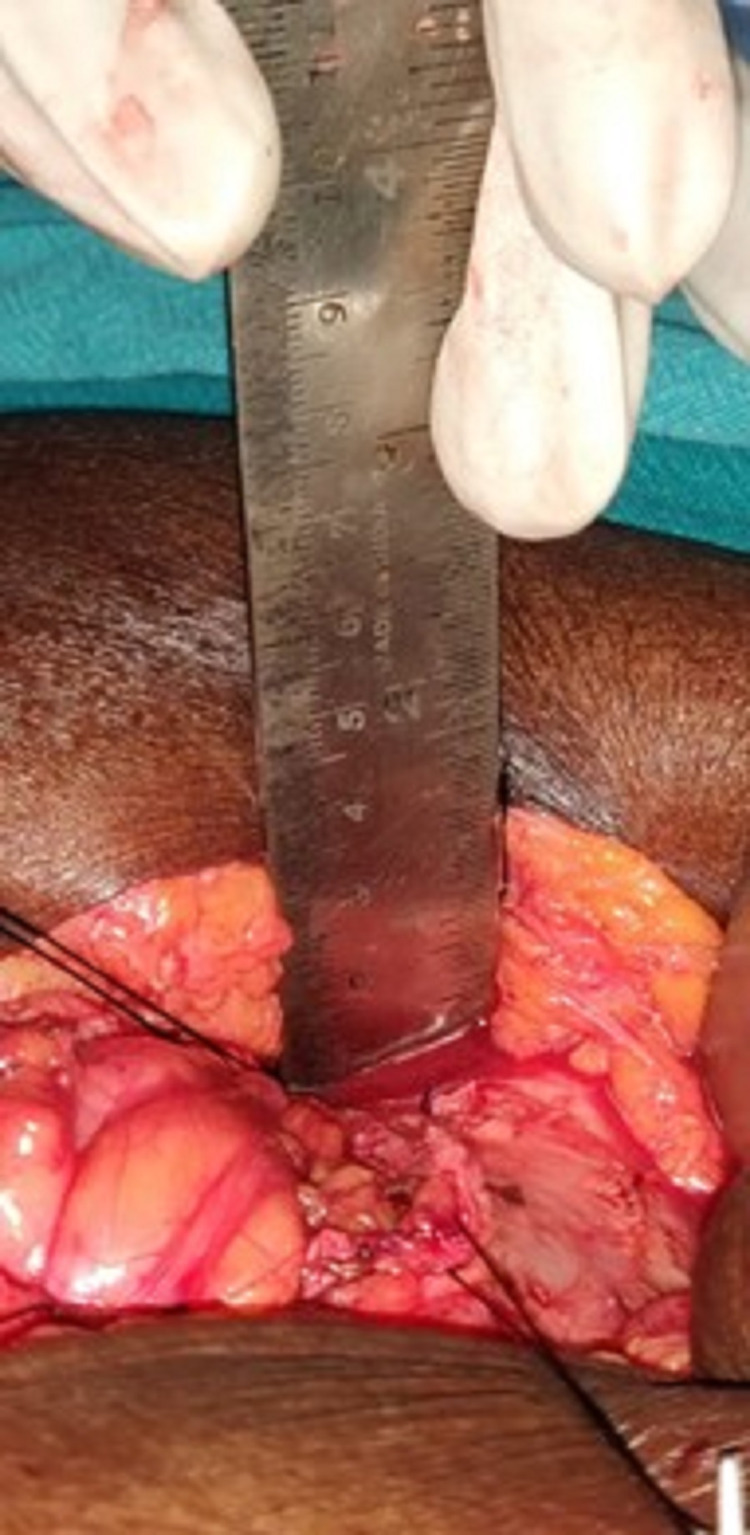
Intraoperative measurement of subcutaneous fat thickness.

Abdominal subcutaneous fat thickness was measured in the supine position intraoperatively with a measuring scale from below dermis to rectus sheath. Midline abdominal subcutaneous fat thickness (SFT) was measured transversely at 1cm caudal to umbilicus level (Figure 1).

Diabetes

Screening for diabetes was done for all patients in the study. For diabetic patients who underwent elective surgeries, preoperative fasting blood glucose level below 120 was achieved. However, for diabetic patients who were operated on emergency basis surgery was done irrespective of the blood glucose levels preoperatively. Postoperative blood glucose levels were monitored for all patients. Insulin was administered to all the patients postoperatively to maintain a blood glucose level below 200mg/dl. Organisms responsible for causing SSI were identified. Any complications occurring in diabetic patients with respect to wound healing or wound dehiscence in an infected wound were recorded.

Obesity

Body mass index (BMI) was calculated for all the patients. BMI was calculated by using the formula BMI = weight in kilograms/height in square meters. Patients with a BMI of more than 30 were taken as obese. Any complications occurring in obese patients with respect to wound healing or wound dehiscence in an infected wound were recorded.

Classification of Surgical Wound

Surgical wounds were classified based on the Centers for Disease Control (CDC) classification as clean, clean-contaminated, contaminated, dirty based upon the degree of contamination. The degree of risk for an SSI is linked to the type of surgical wound.

Statistical analysis

The data were analyzed using IBM SPSS Statistics for Windows, version 27.0 (Armonk, NY: IBM Corp.), and descriptive statistics were expressed as a number and a percentage for categorical variables and as mean standard deviation for quantitative data. Mann-Whitney U test, chi-square test were used to test the continuous and categorical risk factors with the infections. The results were considered statistically significant if the p-value was <0.05 with a 95% confidence interval. To identify the predictors of SSI, univariate and multivariable logistic regression analyses were also performed. Only variables with p< 0.1 in univariate analysis were subjected to multivariable logistic regression analysis. Multicollinearity of the above variables was assessed by calculating variance inflation factor (VIF) with a cut-off value of 5. Nagelkerke's R-square value was used to provide an indication of the amount of variation in the dependent variable explained by the logistic regression model.

## Results

General patient characteristics

Our study cohort consisted of a total of 147 patients. Out of 147 patients, 91 were male and 56 were female. The mean age of the patients in our study was 52.7. The mean duration of surgery was 180 minutes. The mean BMI of the patients was 24.17. The overall incidence of SSI in our study cohort was 10.8% (16 out of 147). General characteristics of the study population are described in Table 1.

**Table 1:** General characteristics of the study population. SSI: surgical site infection; BMI: body mass index

Variables		n (%)	Incidence of SSI, n (%)
Age	<20	3 (2)	0 (0)
	20-40	39 (26.6)	4 (25)
	40-60	48 (32.6)	2 (12.5)
	>60	57 (38.8)	10 (62.5)
Sex	Male	91 (62)	11 (68.8)
	Female	56 (38)	5 (31.2)
Diabetes	Present	43 (29.2)	9 (56.2)
	Absent	104 (70.8)	7 (43.8)
Type of surgery	Elective	77 (52.4)	4 (25)
	Emergency	70 (47.6)	12 (75)
Type of wound	Clean	2 (1.3)	1 (6.2)
	Clean contaminated	83 (56.5)	2 (12.5)
	Contaminated	25 (17)	4 (25)
	Dirty	37 (25.2)	9 (56.3)
BMI	Obese	17 (11.6)	9 (56.2)
	Non-obese	130 (88.6)	7 (43.8)

The mean subcutaneous fat thickness of all the patients in the study was 30.43mm. Fourteen out of 119 (11.76%) patients with fat thickness more than 2.5cm had SSI and two patients out of 29 (6.89%) with subcutaneous fat thickness less than 2.5cm had SSI. Out of 16 patients of SSI 14 patients had subcutaneous fat thickness >2.5cm. Mean TSF in those who developed SSI was 36.4mm. Four patients with wound dehiscence had TSF >2.5mm.

Out of 147 cases, subcutaneous drain was placed in 76 (51.7%) patients. Out of 76 drain groups, six (7.89%) patients developed SSI, and out of 71 no drain groups, 10 (14.08%) patients developed SSI. Out of 16 patients of SSI, six (37%) patients had subcutaneous drain. Ten patients (63%) with no subcutaneous drains in the study developed SSI. Comparison of incidence of SSI among patient groups with various combinations of TSF and drain are presented in Table 2.

**Table 2:** Comparison of incidence of SSI among patient groups with various combinations of TSF and drain. SSI: surgical site infection; TSF: thickness of subcutaneous fat

	Total	SSI	No SSI
Thickness < 2.5cm without drain	26	2 (7.69%)	24 (92.3%)
Thickness < 2.5cm with drain	6	0 (0%)	6 (100%)
Thickness > 2.5cm without drain	45	8 (17.7%)	36 (82.3%)
Thickness > 2.5cm with drain	70	6 (8.5%)	64 (77.14%)

Comparison of clinical characteristics between SSI and no SSI groups

Statistical comparative analysis of various variables between SSI and no SSI groups is presented in Table 3. Both BMI and SFT were significantly associated with SSI, but SFT association was more statistically significant. Other variables with statistically significant association with SSI in our study were diabetes and emergency surgery. Other risk factors associated with SSI like smoking, anemia, and underlying systemic diseases were not evaluated in our study.

**Table 3:** Comparison of variables between SSI and no SSI groups. SSI: surgical site infection; BMI: body mass index Values are reported as n (%), mean ± standard deviation, or median (interquartile range).

Variable	SSI (n = 16)	No SSI (n = 131)	P-value
Age (years)	63.5 (35.75-68)	55 (37-65)	0.370
Male sex	11 (68.8)	80 (61.1)	0.550
BMI	30.7 (22.7-31.1)	23.6 (21.4-25.7)	0.008
Fat thickness (mm)	34.2 (26.9-47.2)	24.1 (21.1-27.4)	0.003
Diabetes	9 (56.3)	34 (26.0)	0.012
Duration of surgery (mins)	185 (125-230)	180 (120-230)	0.685
Emergency surgery	12 (75.0)	58 (44.3)	0.020
Drain	71 (54.2)	6 (37.5)	0.207

Univariate and multivariate analysis

To investigate associations between individual risk factors and incidence of incisional SSI, an initial univariate analysis was performed. Only the factors found to be p<0.1 in univariate analysis were subjected to multivariable analysis. In our study, BMI, TSF, diabetes, and emergency surgery were found to be p<0.1 on univariate analysis. Variance inflation factor (VIF) for diabetes and emergency surgery was less than two. But VIF for BMI (5.9) and TSF (5.8) were more than five, indicating multicollinearity was an issue. Hence, variable with the highest VIF, i.e., BMI was dropped from the final model for multivariate analysis (Table 4). Multivariate analysis showed that TSF was independently associated with SSI (p=0.001). Additionally, emergency nature of surgery was also independently associated with SSI (p= 0.012).

**Table 4:** Final model adjusted for multicollinearity. BMI: body mass index; OR: odds ratio

Variable	Univariate Analysis		Multivariable Analysis	
	OR (95% CI)	P-value	OR (95% CI)	P-value
Age	1.01 (0.98-1.04)	0.504	-	-
Male sex	1.40 (0.46-4.27)	0.552	-	-
BMI	1.22 (1.08-1.38)	0.001		
Fat thickness	1.12 (1.04-1.2)	0.001	1.16 (1.06-1.26)	0.001
Diabetes	3.67 (1.27-10.61)	0.016	2.20 (0.64-6.43)	0.231
Duration of surgery	1.00 (0.99-1.01)	0.715	-	-
Emergency	3.78 (1.16-12.32)	0.028	6.84 (1.52-30.75)	0.012
Drain	0.51 (0.17-1.48)	0.213	-	-

Alternatively, in the model with TSF, Nagelkerke's R-square value was 0.278, whereas in the model with BMI, it was 0.277. Although there was no significant difference between the two models, the higher the value of R-square, the best the model is. The model which is more clinically relatable was used. Hence, there was no statistically significant difference between the independent association of BMI and TSF with SSI on multivariable logistic regression in our study.

## Discussion

The main aim of this prospective study was to estimate the incidence of SSI in patients undergoing abdominal surgeries and analyze various risk factors associated with SSI. The incidence of SSI in our study was 10.8%. The global estimates of SSI have varied from 0.5% to 15%, studies in India have consistently shown higher rates ranging from 23% to 38% [14]. The rates of SSI are much higher with abdominal surgery than with other types of surgery, with several prospective studies indicating an incidence of 15-25% depending on the level of contamination [15]. The lesser incidence in our study can be attributed to the strict institutional policy on the prevention of SSI.

Evaluation of the association between SFT and SSI in patients undergoing laparotomies was the main objective of our study. Cai et al. demonstrated in 246 patients, the mean TSF was 10.87mm, and the cut-oﬀ point for developing SSI was 10.2mm in Crohn's disease patients undergoing laparotomy [9]. Soper et al. reported that only patients with a depth of subcutaneous fat > 3cm developed SSI after undergoing an abdominal hysterectomy [7]. Fujii et al. reported a cut-oﬀ value for TSF of 20mm in Asian patients who developed SSI after undergoing elective colorectal surgery [8]. Most of these studies were either disease-specific or procedure-specific and had variable cut-off values for SFT. Considering the above variations, we used a cut-off value of 2.5cm for patients undergoing laparotomy irrespective of underlying pathology. Our study showed mean subcutaneous fat thickness who developed SSI was 36.4mm which is consistent with previous studies showing increased fat thickness is associated with the development of SSI. The relation between SFT and SSI (p=0.003) was indeed more statistically significant than the relation between BMI and SSI (p=0.008). However, in our study, there was no statistically significant difference between the independent association of BMI and TSF with SSI on multivariable logistic regression due to high multicollinearity. Nevertheless, our study highlighted the relevance of using TSF for the prediction of SSI and is a good addition to the existing literature on this under-explored topic. Another peculiar feature of our study was the method of measurement of SFT. Our method of direct intraoperative measurement is simple and cost-effective when compared to various radiological methods (CT, USG) used in previous studies [8-12]. This method can be reliably used to measure SFT for predicting SSI, especially in low-resource settings.

Another important variable studied was the usage of drain. Many studies have proved that placing subcutaneous drain in laparotomy helps in reducing the incidence of surgical site infection. Gupta et al. reported statistically significant lower incidence of infection in the drain group was lower than the no drain group [16]. Arer et al. demonstrated reduced SSI with subcutaneous suction drains in open abdominal surgery [17]. Fujii et al. showed patients with thick subcutaneous fat and the risk ratio showed a reduction in the SSI rate in the drain group [8]. Imada et al. showed no significant difference in SSI incidence when using a drain in all patients; however, there was a reduction in SSIs in the high-risk patient group from 15% to 8% [18]. In our study, out of 16 patients of SSI, six patients (37%) had subcutaneous drain and 10 (63%) patients belonged to the no drain group. Although the relation between drain and SSI was not statistically significant in our study, usage of drain resulted in a lower incidence if SSI among high-risk patients with SFT > 2.5. It is evident from our study that subcutaneous drains are beneficial in high-risk and/or obese patients but large-scale randomized control studies are needed to prove the association.

Other variables with statistically significant difference (p<0.05) in our study were diabetes and emergency surgery. Diabetes association with SSI was significant (p=0.01) in our study. Other studies by Razavi et al., Cheadle, and Latham et al. have found that diabetes increases the risk of postoperative wound infection [19-21]. Most of the cases (12) who had SSI in our study were operated on an emergency basis. This is consistent with other studies [19,22]. This can be due to the increased number of contaminated cases operated on an emergency basis.

In our study, the greatest number of cases of SSI were between 61 and 70 years of age group. The mean age is 52.72 years. Many studies have shown that SSI is more common in the older age group [23,24]. The relatively more incidence of SSI in the younger age group in our study can be attributed to increased disease burden in young patients who mostly underwent contaminated and dirty surgeries The number of dirty and contaminated surgeries with SSI was more than clean-contaminated surgeries. Findings in various studies have shown that there is a significant rise in infection rate with an increased degree of operative contamination [20,21]. In our study, most cases of SSI occurred in dirty surgeries, which is consistent with the literature.

A major limitation of our study is the study design. A randomized control study with large sample is more persuasive than a simple observational study. Combining elective and emergency surgeries in our study is another major limitation that might have affected our results. There are other known predictors or plausible factors associated with SSI that were not evaluated in this study, including cardiac disease, anemia, malnutrition, and hypoalbuminemia. The comparatively lesser incidence of SSI in our study might have influenced the accuracy of our data. In contrast to previous studies which were either disease-specific or procedure-specific, our study tried to encompass all laparotomies irrespective of pathology. Although a novel approach, generalization of our results requires further randomized studies with large sample. Despite these limitations, our study highlighted the predictive value of SFT as a risk factor for postoperative SSI in patients undergoing laparotomies.

## Conclusions

Our results suggest that the risk of incisional SSI increases with increased subcutaneous fat thickness more than 2.5cm. Placement of subcutaneous drain in patients undergoing laparotomy with increased subcutaneous fat thickness plays a significant role in reducing the incidence of surgical site infection. Delayed wound healing was more in patients with SSI with diabetes. Delayed wound healing and surgical wound dehiscence were more in patients with SSI with obesity. The risk of SSI can be assessed intraoperatively by assessing total subcutaneous fat thickness. Proper use of antibiotics, control of morbidities like diabetes, proper care of obese and thick TSF patients, use of subcutaneous drain in patients with increased fat thickness, and avoidance of cross-infection can help in reducing SSI in patients undergoing abdominal surgeries. Our direct intraoperative method for measuring TSF is a simple and cost-effective tool.
